# Unifying Molecular Weights of Highly Linear Polyethylene Waxes through Unsymmetrical 2,4-Bis(imino)pyridylchromium Chlorides

**DOI:** 10.3390/molecules25235584

**Published:** 2020-11-27

**Authors:** Badral Gansukh, Qiuyue Zhang, Chantsalnyam Bariashir, Arumugam Vignesh, Yanping Ma, Tongling Liang, Wen-Hua Sun

**Affiliations:** 1 Key Laboratory of Engineering Plastics and Beijing National Laboratory for Molecular Sciences, Institute of Chemistry, Chinese Academy of Sciences, Beijing 100190, China; baagii11a@iccas.ac.cn (B.G.); zhangqiuyue@iccas.ac.cn (Q.Z.); bariashir@iccas.ac.cn (C.B.); avignesh@iccas.ac.cn (A.V.); ltl@iccas.ac.cn (T.L.); 2 CAS Research/Education Center for Excellence in Molecular Sciences, University of Chinese Academy of Sciences, Beijing 100049, China; 3 State Key Laboratory for Oxo Synthesis and Selective Oxidation, Lanzhou Institute of Chemical Physics, Chinese Academy of Sciences, Lanzhou 730000, China

**Keywords:** *ortho*-fluorinated chromium pre-catalysts, ethylene polymerization, highly linear PE waxes, single-site catalysis

## Abstract

By dealing CrCl_3_∙3THF with the corresponding ligands (**L1**–**L5**), an array of fluoro-substituted chromium (III) chlorides (**Cr1**–**Cr5**) bearing 2-[1-(2,4-dibenzhydryl-6-fluoro- phenylimino)ethyl]-6-[1-(arylimino)ethyl]pyridine (aryl = 2,6-Me_2_Ph **Cr1**, 2,6-Et_2_Ph **Cr2**, 2,6-iPr_2_Ph **Cr3**, 2,4,6-Me_3_Ph **Cr4**, 2,6-Et_2_-4-MePh **Cr5**) was synthesized in good yield and validated via Fourier Transform Infrared (FT-IR) spectroscopy and elemental analysis. Besides the routine characterizations, the single-crystal X-ray diffraction study revealed the solid-state structures of complexes **Cr2** and **Cr4** as the distorted-octahedral geometry around the chromium center. Activated by either methylaluminoxane (MAO) or modified methylaluminoxane (MMAO), all the chromium catalysts exhibited high activities toward ethylene polymerization with the MMAO-promoted polymerizations far more productive than with MAO (20.14 × 10^6^ g (PE) mol^−1^ (Cr) h^−1^ vs. 10.03 × 10^6^ g (PE) mol^−1^ (Cr) h^−1^). In both cases, the resultant polyethylenes were found as highly linear polyethylene waxes with low molecular weights around 1–2 kg mol^−1^ and narrow molecular weight distribution (MWD range: 1.68–2.25). In general, both the catalytic performance of the *ortho*-fluorinated chromium complexes and polymer properties have been the subject of a detailed investigation and proved to be highly dependent on the polymerization reaction parameters (including cocatalyst type and amount, reaction temperature, ethylene pressure and run time).

## 1. Introduction

**Chart 1:**
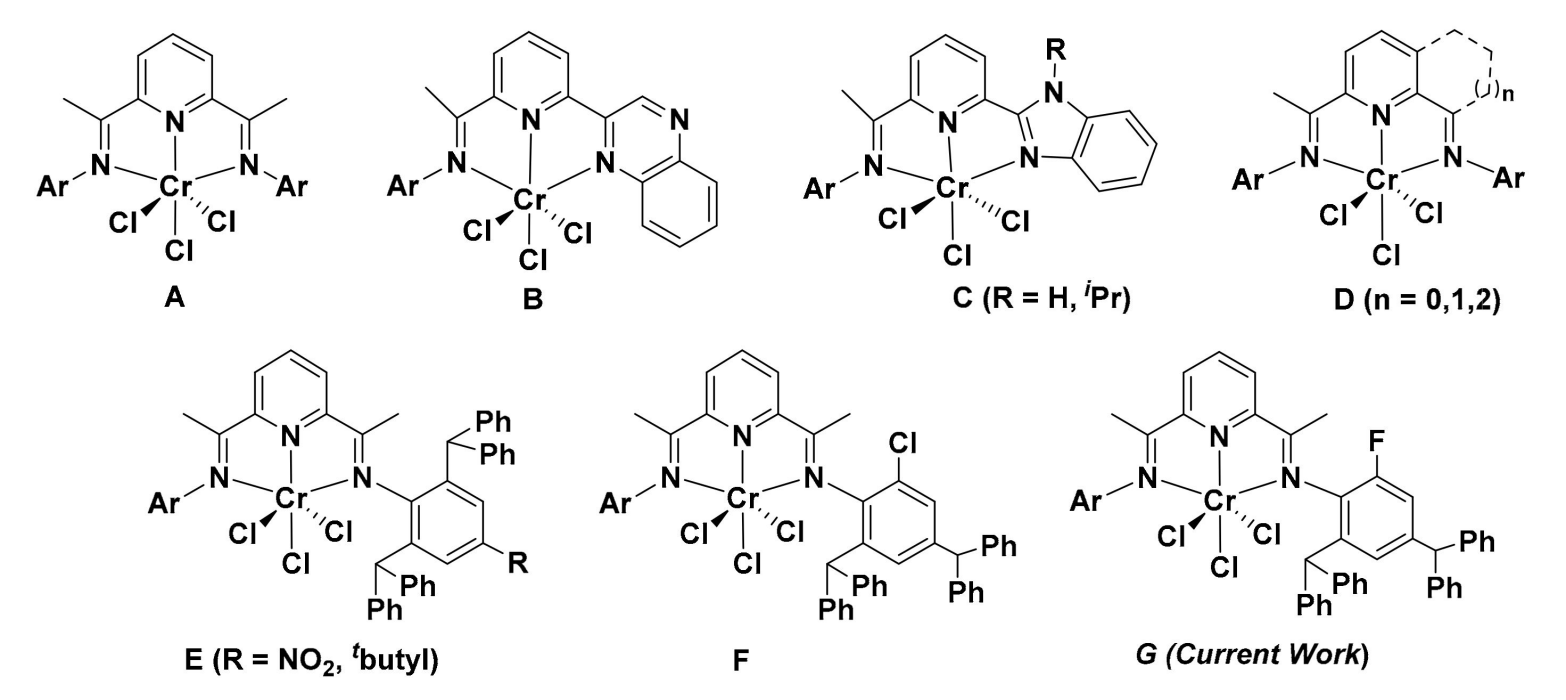
Chromium (III) pre-catalysts (**B**–**G**) derived from 2,6-bis(arylimino)pyridine-containing (**A**).

Since silica-supported Phillips [1,2,3,4,5] and Union Carbide [6,7,8] catalyst systems became extensively used in commercial production of polyolefins, the development of Cr-based catalysts has been a critical research issue for researchers [9,10,11,12,13,14,15,16,17,18,19,20,21,22,23,24,25,26,27,28,29,30,31]. Many efforts have been made to expanded the type of heterogeneous and homogeneous catalysts based on chromium for ethylene oligomerization, polymerization [13,14,15,16,17,18,19,20,21,22,23,24,25] as well as for ethylene trimerization [26,27,28,29,30] and tetramerization [31]. As homogeneous catalysts allow the production of polymers with narrow molecular weight distributions compared to heterogeneous ones [32,33,34,35,36,37], the exploration of promising homogeneous chromium precatalysts for ethylene oligomerization and/or polymerization by exploiting new ancillary ligands framework has gradually become one of the hot issues of research in this field [3,14,19,21,23,38]. Subsequently, on the basis of the classical model of 2,6-bis(imino)pyriylchromium (III) precatalysts (**A**, Chart 1) [23,39,40,41], numerous structural modifications have been made to improve the 2,6-diiminopyridine framework including 2-quinoxalinyl-6-iminopyridines (**B**, Chart 1) [42], 2-benzimidazolyl-6-(1-(arylimino)ethyl)pyridines (**C**, Chart 1) [43,44] and cycloalkyl-fused bis(arylimino)pyridines (**D**, Chart 1) [45,46,47,48] as well as their derivatives [49,50,51,52,53]. The chromium complexes **B** and **C** (Chart 1) and their derivatives showed moderate or high activities towards ethylene oligo-/polymerization generating oligomers or a mixture of oligomers and polyethylene waxes [42,43,44,49,50,51], while all the cycloalkyl-fused bis(arylimino)pyridylchromium (III) complexes (**D**, Chart 1) were able to produce strictly linear polyethylenes or vinyl-terminated polyethylene waxes [52]. In addition, functionalizing *ortho*-position of *N*-aryl groups with bulky dibenzhydryl substituents can increase the steric demand of the whole ligand set and shield the apical positions of the coordination-planar metal centers [54,55,56,57,58,59,60,61]. Consequently, 2-(1-(2,6-dibenzhydryl-4-*R*-phenyl imino)-ethyl)-6-(1-(arylimino)- ethyl)pyridylchromium (III) precatalysts (R = NO_2_, *t*-butyl) (**E**, Chart 1) were reported to exhibit moderate activities toward ethylene polymerization while produce high-molecular-weight linear polyethylenes [62,63]; thereinto, nitro-enhanced 2,6-bis(imino)pyridylchromium (III) chlorides had a definite advantage in increasing catalytic activity and molecular weight over *t*-butyl-functionalized 2,6-bis(imino)pyridylchromium (III) chlorides.

Inspired by the positive result brought by the nitro electron-withdrawing group, the halogen atom has been introduced to the *ortho*-position of *N*-aryl groups (**F**, Chart 1) to enhance their catalytic performance of bis(imino)pyridyl chromium catalysts. Surprisingly, the *ortho*-chloro-substituted 2-[1-(2,4-dibenzhydryl-6-chlorophenylimino)ethyl]-6-[1-(arylimino)ethyl]- pyridylchromium (III) chloride precatalysts [64] were found to display high activity (up to 14.96 × 10^6^ g (PE) mol^−1^ (Cr) h^−1^ at 60 °C) affording highly linear polyethylene with moderate molecular weight (M_w_) ranging from 4.01 to 22.06 kg mol^−1^. With a view to further explore the effect of *ortho*-fluoro group with stronger electron-withdrawing ability on catalytic performance of chromium catalysts, herein, we report the synthesis route and characterization data of the *ortho*-fluoro substituted 2,4-bis(imino)pyridylchromium (III) chloride complexes (**G**, Chart 1) along with their ethylene polymerization behavior. A detailed catalytic evaluation of these chromium catalysts was performed using methylaluminoxane (MAO) and modified methylaluminoxane (MMAO) as cocatalysts to identify the most suitable polymerization conditions. Moreover, the correlation between the properties of resultant polymers and the electronic and steric effect of the ligand framework as well as the reaction parameters will be discussed at length.

## 2. Results

### 2.1. Synthesis and Characterization

**Scheme 1:**
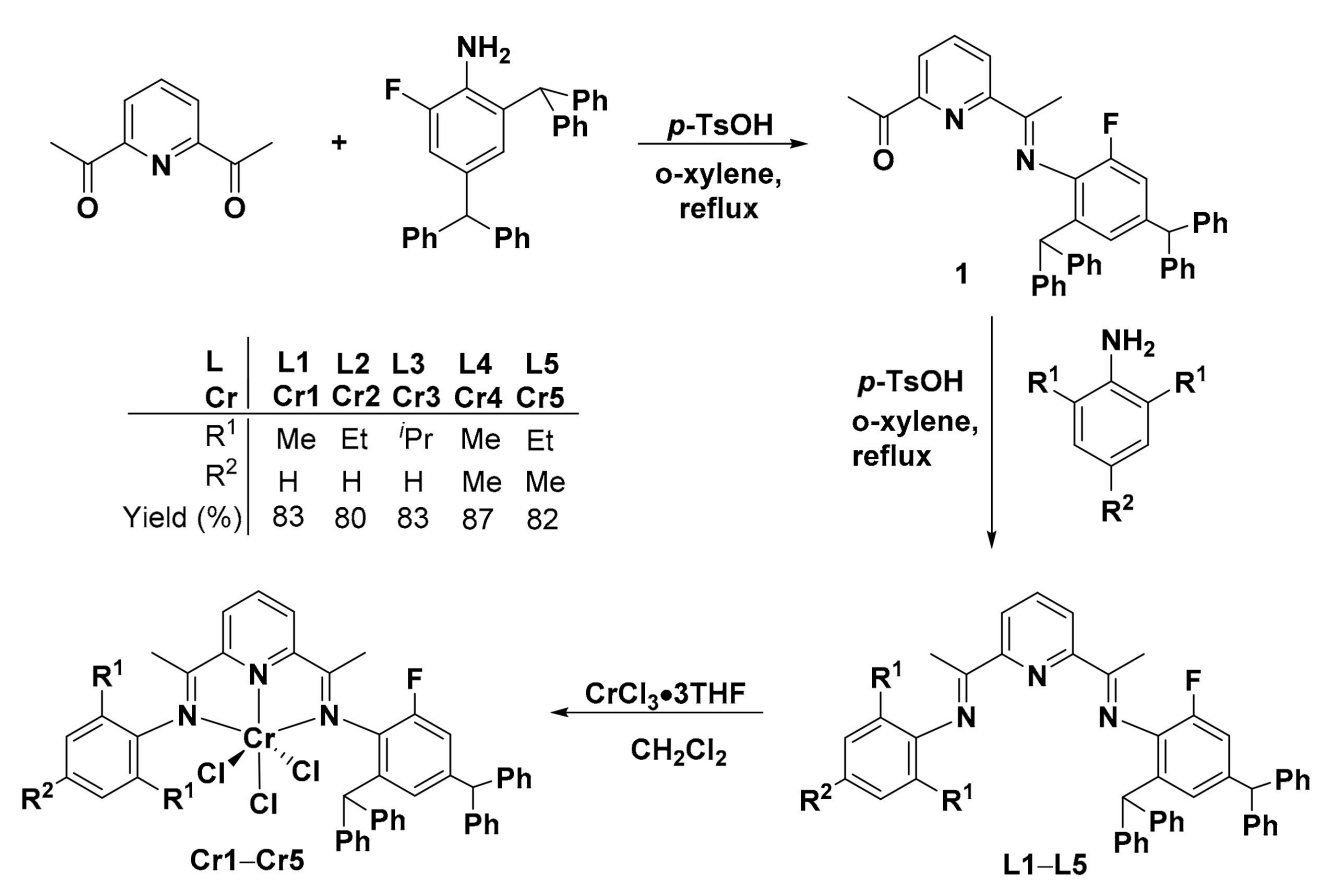
Synthesis of ligands **L1**–**L5** and the corresponding chromium complexes (**Cr1**–**Cr5**).

The diiminopyridine derivatives (**L1**–**L5**) were prepared in moderate yields by a two-step procedure (Scheme 1) [62,63,64] and confirmed by various characterization methods including ^1^H/^13^C Nuclear Magnetic Resonance (NMR), FT-IR spectra and elemental analysis [49,55,57]. The stoichiometric reactions of the diiminopyridine compounds (**L1**–**L5**) with CrCl_3_·3THF in dichloromethane being stirred for 10 h under room temperature gave corresponding 2-[1-(2,4-dibenzhydryl-6-fluorophenyl- imino)ethyl]-6-[1-(arylimino)ethyl]pyridylchromium (III) chlorides [aryl = 2,6-Me_2_C_6_H_3_ (**Cr1**), 2,6-Et_2_C_6_H_3_ (**Cr2**), 2,6-*^i^*Pr_2_C_6_H_3_ (**Cr3**), 2,4,6-Me_3_C_6_H_2_ (**Cr4**), 2,6-Et_2_-4-MeC_6_H_2_ (**Cr5**) in good yields (80–87%). The FTIR spectra of these chromium complexes show that the ν(C=N)_imine_ stretching frequencies fell in the range 1611–1618 cm^−1^ which compared to 1638–1643 cm^−1^ for the free ligands indicating an effective coordination between the imine-nitrogen and the chromium metal. The coordination of chromium with nitrogen atom in *ortho*-chloro substituted chromium (III) complexes can cause less redshifts (around 21 cm^–1^) of the C=N absorption band than that (around 26 cm^–1^) in *ortho*-floro substituted chromium (III) complexes a result of different donor structure [64]. Moreover, the molecular structures of **Cr2** and **Cr4** were further confirmed by the single-crystal X-ray diffraction.

### 2.2. X-ray Crystallographic Studies

**Figure 1:**
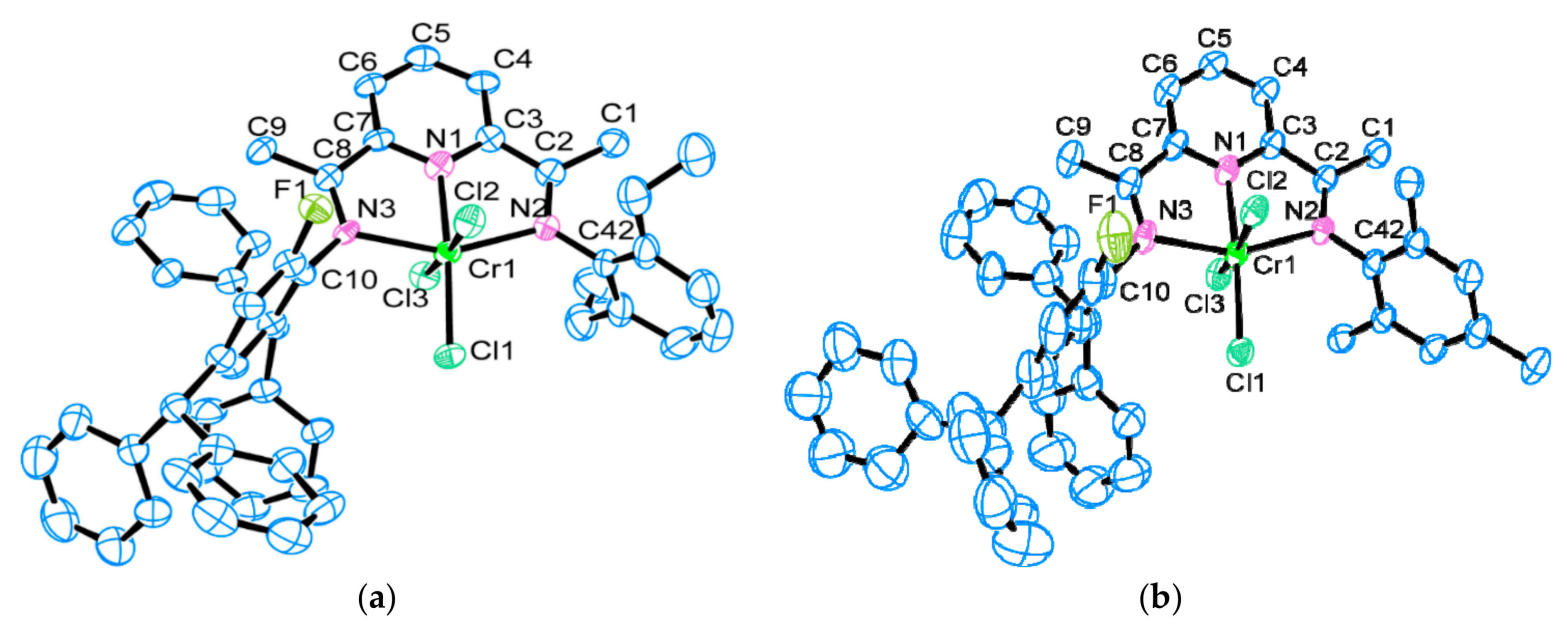
ORTEP drawing of **Cr2** (**a**) and **Cr4** (**b**) with thermal ellipsoids set at a 30% probability level. For clarity purposes, all H atoms are omitted in both cases.

Single-crystals of the complexes **Cr2** and **Cr4** suitable for the X-ray determination were individually grown by the slow diffusion of *n*-heptane into their respective dichloromethane solutions. The Oak Ridge Thermal Ellipsoid Plot (ORTEP) diagrams of **Cr2** and **Cr4** are presented in Figure 1, respectively while the selected bond lengths and angles of **Cr2** and **Cr4** are listed in Table 1. These two complexes have similar coordination geometry hence they will be discussed together. Complexes **Cr2** and **Cr4** were mononuclear species in which chromium center coordinated with three chloride atoms forming a six-coordinate geometry described as a distorted-octahedral geometry. These three chloride ligands were disposed in a mer arrangement. The Cl1, N1, N2, and N3 atoms constituted the equatorial plane while two axial bonds nearly formed a linear through the chromium center [Cl(1)-Cr(1)-Cl(3), 91.04(8)° for **Cr2** and 93.74(4)° for **Cr4,** respectively]. The bond length of Cr-N_pyridine_ (Cr1-N1, 2.003(5) Å for **Cr2** and 1.985(3) Å for **Cr4**) was evidently shorter than that of corresponding Cr–N_imino_ (Cr1-N2, 2.157(5) Å and Cr1-N3, 2.133(5) Å for **Cr2**; Cr1-N2, 2.132(3) Å and Cr1-N3, 2.136(3) Å for **Cr4**) highlighting that a stronger bond between the pyridine donor and the metal center was present than that between the imine-nitrogen and the metal center. Compared to the *ortho*-chloro substituted chromium (III) complexes, in this work the Cr-N_pyridine_ bond length was longer [2.003(5) Å vs. 1.994(2) Å for **Cr2** in both cases] while Cr-Cl and imine bond lengths were generally shorter due to the halogen effects [64]. Furthermore, the obvious deviation of bond lengths of the two Cr-N_imino_ bonds (Cr1-N2 and Cr1-N3) in **Cr2** and **Cr4** was mainly due to the unsymmetrical framework consistent with the observations of their analogs [49,50,51,52,53,62,63,64]. The bond lengths of the two imine nitrogen atoms in **Cr4** were also distinct (1.297(5) for C2-N2 and 1.2817(6) for C8-N3), potentially due to different steric properties of *N*-aryl groups [62,63].

### 2.3. Ethylene Polymerization

To identify a suitable polymerization condition that can be used to evaluate all the five chromium pre-catalysts (**Cr1**–**Cr5**) for the polymerization of ethylene, **Cr4** was chosen as the test precatalyst in the first instance to allow an optimization of various catalytic parameters. Based on previous studies of structurally related *N,N,N*-bound chromium(III) complexes [49,50,51,52,53,64], methylaluminoxane (MAO) and modified methylaluminoxane (MMAO) have proved the most effective co-catalysts to promote ethylene polymerization. Hence, these two cocatalysts were employed to activate the chromium precatalysts and the optimum reaction conditions including run temperature, Al/Cr molar ratio, reaction time and ethylene pressure were separately ascertained for the catalytic system composed of **Cr4**/MAO and **Cr4**/MMAO.

#### 2.3.1. Catalytic Evaluation of Cr4/MAO Catalytic System

**Figure 2:**
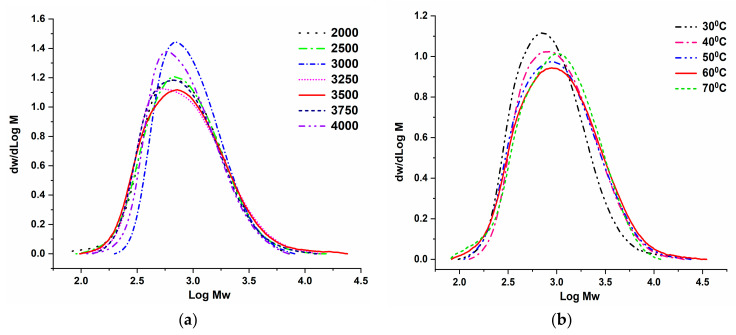
(**a**) GPC curves for the polyethylene obtained using **Cr4**/MAO at various Al/Cr ratios with the reaction temperature fixed at 30 °C (entries 1–7, Table 2); (**b**) GPC curves of the polyethylene formed using **Cr4**/MAO at different temperatures with the Al/Cr molar ratio fixed at 3500 (entries 5 and 8–11, Table 2).

With the reaction temperature fixed at 30 °C, variation of the molar ratio Al/Cr from 2000 to 4000 was investigated (entries 1–7, Table 2). As the Al/Cr molar ratio was increased, the catalytic activity reached a maximum of 5.46 × 10^6^ g (PE) mol^−1^ (Cr) h^−1^ at the Al/Cr ratio of 3500 (entry 5, Table 2). The Gel Permeation Chromatography (GPC) data generally revealed a narrow and unimodal polydispersity (M_w_/M_n_ range = 1.46–1.89) for the polyethylene formed (Figure 2a); moreover, there was no clear effects shown by the amount of co-catalyst on the molecular weight with very similar values observed across the ratio range (*M_w_* = 1.04–1.22 kg mol^−1^). Interestingly, the Melting temperature (T_m_) value of the resultant polymers followed a similar trend with their corresponding molecular weights (Table 2), a finding that indicates the highly linear properties of the obtained polymers [64].

On varying the polymerization temperature from 30 to 70 °C with the Al/Cr molar ratio fixed at 3500 and the reaction time for 30 min (entries 5, 8–11, Table 2), a peak in catalytic activity was achieved of 10.03 × 10^6^ g (PE) mol^−1^ (Cr) h^−1^ at 60 °C. Further raising the temperature to 70 °C led to a rapid decrease in its activity from 10.03 × 10^6^ g (PE) mol^−1^ (Cr) h^−1^ to 6.24 × 10^6^ g (PE) mol^−1^ (Cr) h^−1^ (Figure 2b), which can be ascribed to the partial deactivation of the active species resulting from increased chain transfer to aluminum at the higher temperature [65,66,67,68,69] and the lower solubility of ethylene in toluene at elevated temperatures [70,71,72]. Although the molecular weights obtained at different temperatures have little difference, the highest molecular weight (1.61 kg mol^−1^) obtained at the optimum temperature 60 °C somehow reflects more probability of chain propagation at this temperature [57].

**Figure 3:**
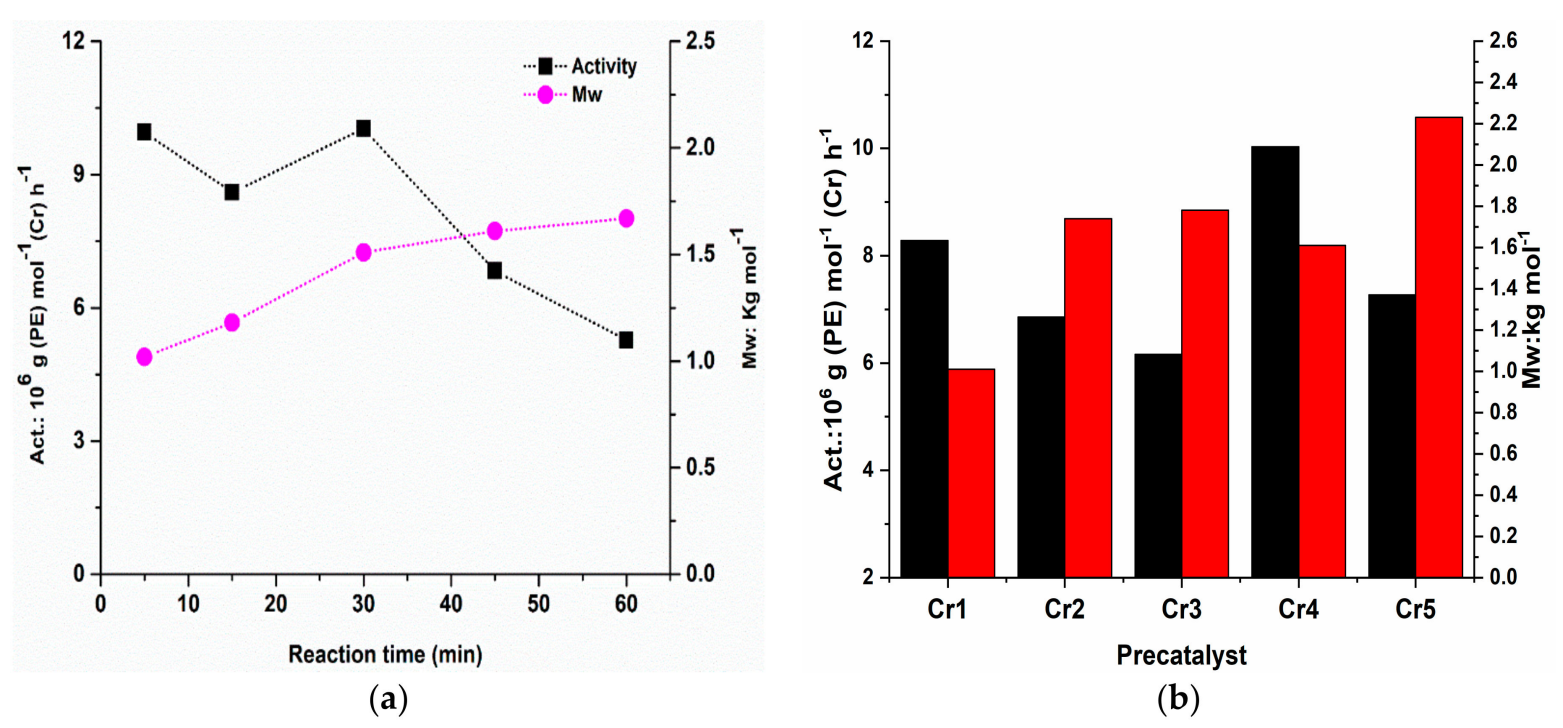
(**a**) Activity and M_w_ vs. reaction time for **Cr4**/MAO system (entries 10 and 12–15, Table 2); (**b**) Comparative activity of **Cr1**–**Cr5** and M_w_ of the corresponding polymers (Table 3).

To investigate the lifetime of the active species in the **Cr4**/MAO system, the catalytic screens were conducted over time (5, 15, 30, 45 and 60 min) with the reaction temperature maintained at 60 °C and the Al/Cr molar ratio of 3500 (entries 10 and 12–15, Table 2). The highest activity of 10.03 × 10^6^ g (PE) mol^−1^ (Cr) h^−1^ was observed at the 30 min mark (entry 10, Table 2) while this activity value was similar with that obtained at 5 and 15 min indicating no obvious induction period needed to generate the active species. Then the catalytic activity gradually decreased with the reaction time extension reaching its lowest value of 5.27 × 10^6^ g (PE) mol^−1^ (Cr) h^−1^ at 60 min (entry 15, Table 2) suggesting that the active species formed slowly after the addition of MAO and underwent progressive deactivation over time [49,50,51,52,53,62,63,64]. While the obtained polyethylene had increased molecular weight over time illustrating that there were sufficient active species present to maintain chain propagation despite gradual deactivation (Figure 3a) [49,50,51,52,53]. With other reaction parameters maintained at the optimum values, on lowering the ethylene pressure from 10 to 5 atm the catalytic activity has more than halved (entry 16 vs. entry 10, Table 2). Further reducing the ethylene pressure to 1 atm, only a trace amount of polymer was obtained (entry 17, Table 2), which is in accord with the previous observations for structurally related chromium pre-catalysts [62,63,64]. These results manifest that high pressure of ethylene is necessary to achieve satisfactory activities consistent with the direct correlation between catalytic activity and ethylene concentration [54,55,56,57,58,59,60,61,62,63,64].

#### 2.3.2. Ethylene Polymerization with the **Cr1**–**Cr5**/MAO Using Optimal Reaction Conditions

With an aim to investigate the influence of structural variations made to the chromium precatalysts on catalyst performance and polymer properties, the remaining four pre-catalysts were investigated for ethylene polymerization under the optimal conditions (Al/Cr = 3500, run temperature = 60 °C, run time = 30 min) established by **Cr4**/MAO (entry 10, Table 2). The activity of these catalysts (**Cr1**–**Cr5**) and molecular weight of the obtained polymers were described in Figure 3b. High activities in the range of (6.16–10.03) × 10^6^ g (PE) mol^−1^ (Cr) h^−1^ were observed with the following order: **Cr4** [2,4,6-tri(Me)] > **Cr1** [(2,6-di(Me)] > **Cr5** [2,6-di(Et)-4-Me)] > **Cr2** [(2,6-di(Et)] > **Cr3** [(2,6-di(*^i^*Pr)] (Table 3), which indicates the catalytic activity closely related with both steric and electronic effects imparted by the second *N*-aryl imine group. **Cr3** containing bulky 2,6-diisopropyl-imine groups was found to exhibit lower catalytic activity as the crowded space around the chromium center led to lower ethylene coordination and insertion rates [57,62,63,73]. The electronic effect was reflected in the presence of an electron-donating *para*-methyl substituent of **Cr4** and **Cr5,** which was beneficial to the improvement of catalytic activity when compared with *para*-hydrogen substituted **Cr1** and **Cr2**, respectively [63]. By comparison with polymerization data recorded for *ortho*-chloro-substituted chromium complexes, all the chromium complexes in this work generally showed higher catalytic activity producing polyethylene with lower molecular weight [64].

**Figure 4:**
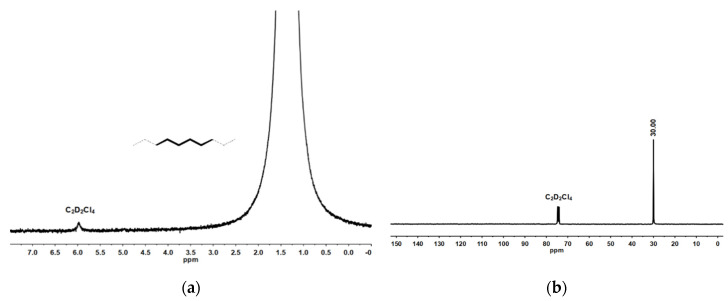
(**a**) ^1^H-NMR spectrum of the polyethylene obtained by **Cr4**/MAO at 60 °C, recorded in 1,1,2,2-tetrachloroethane-*d*_2_ at 100 °C; (**b**) ^13^C-NMR spectrum of the polyethylene obtained by **Cr4**/MAO at 60 °C, recorded in 1,1,2,2-tetrachloroethane-*d*_2_ at 100 °C (entry 10, Table 2).

To investigate the microstructural properties of the polyethylenes generated using **Cr4**/MAO, both DSC and high temperature ^1^H and ^13^C NMR spectroscopic measurements were employed. T_m_ values of the resultant polymers exceeding 120 °C indicates the obtained high-density polyethylene possessed highly linear structures (Table 2 and Table 3). For further confirming this speculation, a representative sample with the highest yield obtained by **Cr4**/MAO at 60 °C (entry 10, Table 2) was subjected to the ^1^H and ^13^C high-temperature spectroscopy (recorded in 1,1,2,2-tetrachloroethane-*d*_2_ at 100 °C) (Figure 4). The prominent singlets at δ =1.35 in the ^1^H spectrum and δ = 30.00 in the ^13^C spectrum corresponding to the repeating -(CH_2_)_n_- repeat units again reflected the strict linearity of resultant polyethylene [49,50,51,52,53]. However, there was no evidence of double bonds present in the chain of polymer meaning that no unsatarated polymer formed along a termination pathway involving β-hydrogen elimination or transfer [51].

#### 2.3.3. Catalytic Evaluation of **Cr4**/MMAO Catalytic System

**Figure 5:**
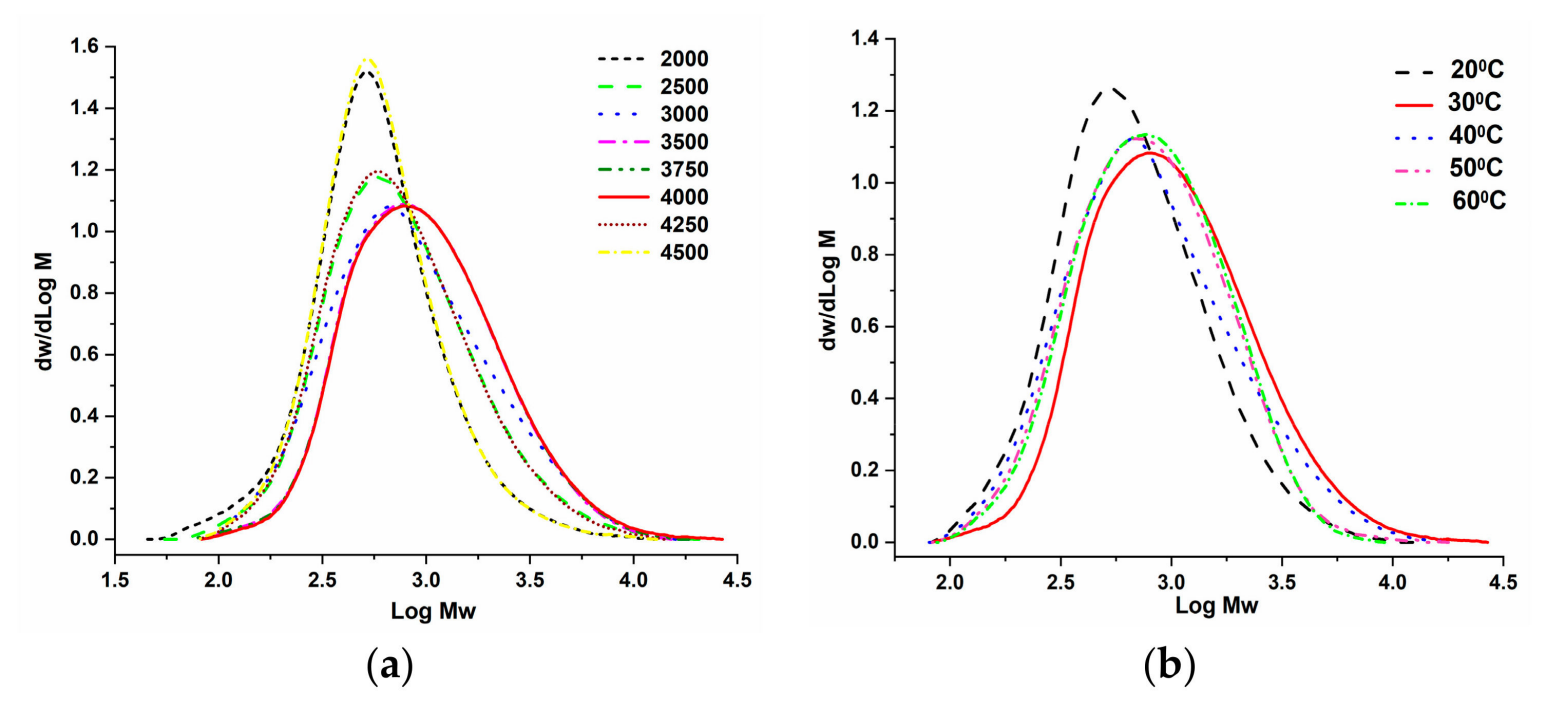
(**a**) GPC curves for the polyethylene obtained using **Cr4**/MMAO at various Al/Cr ratios with the reaction temperature fixed at 30 °C (entries 1–8, Table 4); (**b**) GPC curves of the polyethylene formed using **Cr4**/MMAO at different temperatures with the Al/Cr molar ratio fixed at 4000 (entries 6 and 9–12, Table 4).

To complement the study performed with MMAO as co-catalyst; the results are collected in Table 4. Once again, **Cr4** was chosen as the test pre-catalyst to allow an optimization of the polymerization parameters. The polymerization was conducted at 10 atm of ethylene pressure, and the screening results are given in Table 4. Initially, on increasing Al/Cr molar ratio from 2000 to 4500 at 30 °C, a maximum activity of 20.14 × 10^6^ g (PE) mol^−1^ (Cr) h^−1^ was found with an Al/Cr ratio of 4000 (entry 6, Table 4). **Cr4**/MMAO generates ca. 3.5-fold higher activity in comparison to **Cr4**/MAO, similar to observations reported elsewhere [74]. When further raising the amount of co-catalyst, the activity was sharply reduced to 11.14 × 10^6^ g (PE) mol^−1^ (Cr) h^−1^ as a result of the increased chain transfer from the chromium center to aluminum [61]. In addition, the molecular weight distribution (*M_w_/M_n_* range = 1.62–2.14) remained particularly narrow and unimodal, as shown by the GPC curves (Figure 5a).

With the Al/Cr molar ratio kept at 4000 and the run time set at 30 min, the reaction temperature had been increased from 20 to 60 °C (entries 6 and 9–12, Table 4). The best catalytic activity was attained at 30 °C with a value of 20.14 × 10^6^ g (PE) mol^−1^ (Cr) h^−1^. The differences in optimum polymerization temperature between **Cr**/MAO and **Cr**/MMAO catalytic system may be attributed to the different energy barrier for cocatalysts to activate the chromium precatalyst [56]. At higher temperature, the activity was decreased slowly due to the partially deactivation of the active species [65,66,67,68,69] and lower solubility of ethylene [70,71,72] (Figure 5b) but nevertheless revealed a good level of 11.76 × 10^6^ g (PE) mol^−1^ (Cr) h^−1^ at 60 °C (entry 12, Table 4). Similar to MAO case, the molecular weight of obtained polyethylene reached a peak up to 1.43 kg mol^−1^ at the optimum temperature which indicates that chain propagation served as the dominant reaction before reaching the optimum temperature (≤30 °C) but when further increasing the reaction temperature the higher rate of chain termination resulted in the lower molecular weight of polyethylene [57,65,66,67,68,69].

**Figure 6:**
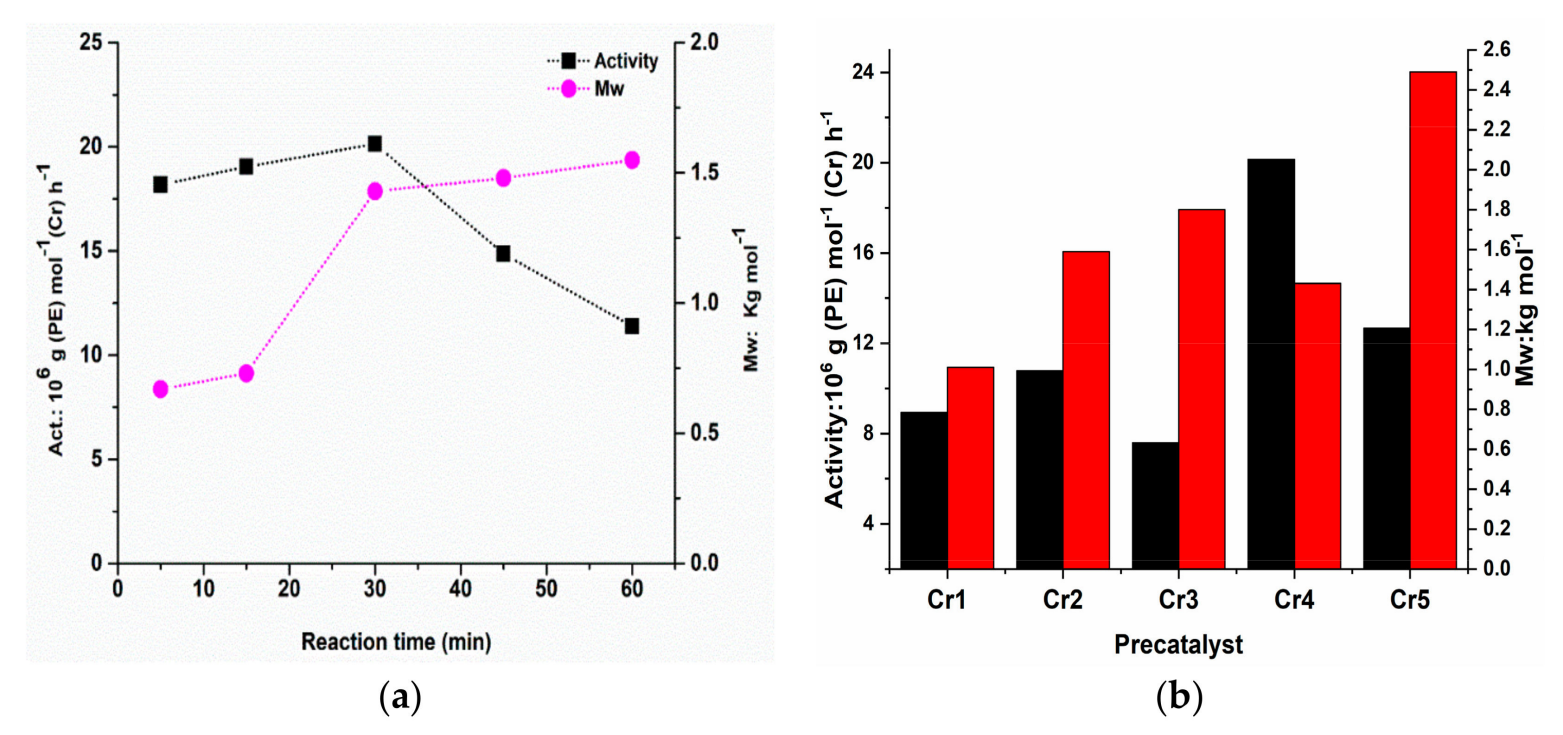
(**a**) Activity and M_W_ vs. reaction time for **Cr4**/MMAO system (entries 6 and 13–16, Table 4); (**b**) Comparative activity of **Cr1**–**Cr5** and M_W_ of the corresponding polymers (Table 5).

In the next step, the temperature was kept at 30 °C and the Al/Cr molar ratio at 4000, the effect of time was investigated by conducting the polymerizations using **Cr4**/MMAO at 5, 15, 30, 45 and 60 min intervals (entries 6 and 13–16, Table 4) (Figure 6a). Similar with the **Cr4**/MAO system, the optimal activity of 20.14 × 10^6^ g (PE) mol^−1^ (Cr) h^−1^ was again achieved within 30 min (entry 6, Table 4). Between 5 to 30 min the activity was gradually increased, while during the second 30 min it was slowly decreased with the onset of catalyst deactivation [49,50,51,52,53,62,63,64]. With regard to molecular weight of the resultant polymers, longer reactions were accompanied by an increase in the value of molecular weight from 0.67 to 1.55 kg mol^−1^; this observation can be attributed to stable presence of sufficient active species over longer reaction time during the polymerization process [57]. Reducing the ethylene pressure was also significantly affected the catalytic performance, which was demonstrated by the much lower activity at 5 atm of C_2_H_4_ and only traced amounts of the polymer were gained at 1 atm of ethylene (entries 17 and 18, Table 4). Additionally, the molecular weight of the obtained polymer at 5 atm of ethylene pressure was lower than that achieved at 10 atm of ethylene pressure, which can be attributed to the slower propagation rate at lower ethylene pressure (entries 6, 17 and 18, Table 4) [54,55,56,57,58,59,60,61,62,63,64].

#### 2.3.4. Ethylene Polymerization with the **Cr1**–**Cr5**/MMAO Using Optimal Reaction Conditions

Using the favored operating conditions established using **Cr4**/MMAO (Al/Cr = 4000, run temperature = 30 °C, run time = 30 min), the remaining precatalysts, **Cr1**–**Cr3** and **Cr5**, were all evaluated using MMAO as cocatalyst (Table 5). According to the data, all the chromium complexes (**Cr1**–**Cr5**) exhibited activities in the range of 7.59–20.14 × 10^6^ g (PE) mol^−1^ (Cr) h^−1^ (Table 5) which were generally higher when compared to **Cr**/MAO catalytic system (6.16–10.03 × 10^6^ g (PE) mol^−1^ (Cr) h^−1^) (Table 3) highlighting the importance of the aluminoxane activator. The overall activity decreased in the order **Cr4** [2,4,6-tri(Me)] > **Cr5** [2,6-di(Et)-4-Me] > **Cr2** [2,6-di(Et)] > **Cr1** [2,6-di(Me)] > **Cr3** [2,6-di(*^i^*Pr)] as a result of the combined action of electronic and steric effects of the ligands (Figure 6b) [57,62,63,73]. By way of comparison, structurally related chromium precatalysts bearing 2,4-dibenzhydryl-6-chlorophenyl groups displayed relatively lower activity while the most hindered **Cr3** showed lower activity than that in this work (Chart 1) indicating that the solubility of catalyst also affected their catalytic activity [64].

**Figure 7:**
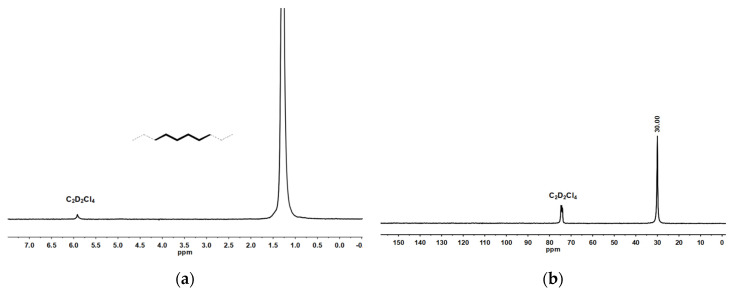
(**a**) ^1^H-NMR spectrum of the polyethylene obtained by **Cr4**/MMAO at 60 °C, recorded in 1,1,2,2-tetrachloroethane-*d*_2_ at 100 °C; (**b**) ^13^C-NMR spectrum of the polyethylene obtained by **Cr4**/MMAO at 60 °C, recorded in 1,1,2,2-tetrachloroethane-*d*_2_ at 100 °C.

To further study the effect of cocatalyst type on the microstructures of the polymers, the sample achieved by **Cr4/**MMAO at 30 °C (entry 6, Table 4) was also measured by high temperature ^1^H-NMR and ^13^C NMR spectroscopic study. Similar with the result in MAO case, the presence of singlet resonances in both the ^1^H-NMR spectrum (at δ 1.35, Figure 7a) and the ^13^C-NMR spectrum (at δ 30.0, Figure 7b) is characteristic with high linearity polyethylene, corresponding to the methylene (-CH_2_-) repeat unit, again confirmed the formation of highly linear polyethylene which was further corroborated by its high melting temperature (T_m_ > 119 °C) (Figure 7) [49,50,51,52,53].

**Figure 8:**
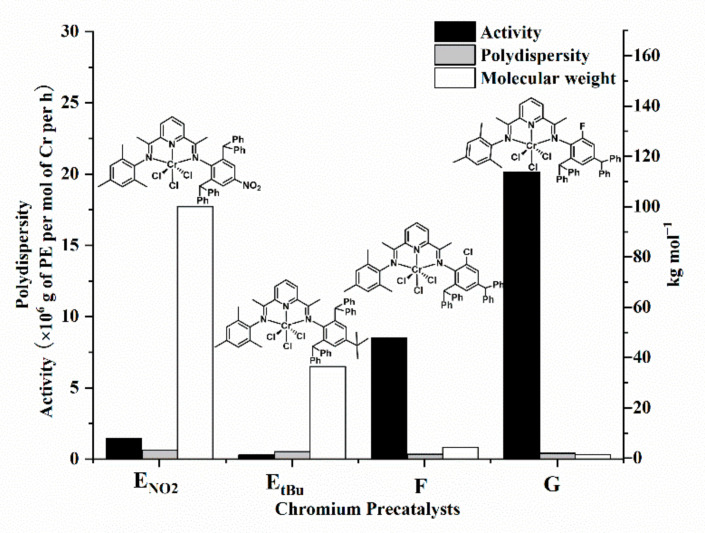
Comparative catalytic performance of **Cr4** (**G** in Chart 1) (entry 4, Table 5) with structrually related chromium-containing **E_NO2_** [62], **E_tBu_** [63] and **F** [64] (Chart 1); all polymerizations were recorded at 10 atm C_2_H_4_, 30 min using MMAO as co-catalyst.

To allow a comparison of these current chromium precatalysts (**G** in Chart 1) with the structurally related chromium systems, **E** and **F** (Chart 1), the optimum activity and the molecular weight as well as the polydisperisity of resultant polymers observed for each precatalyst are depicted in Figure 8. All polymerization tests were performed under their optimum condition at 10 atm C_2_H_4_ over 30 min using MMAO as cocatalyst [62,63,64]. Inspection of Figure 8 shows that **G** exhibited the highest catalytic activity of all four classes highlighting the beneficial effect of strong electro-withdrawing group (*ortho*-fluoro substitution) on improving catalytic activity of precatalysts [75]. Moreover, the chromium precatalysts **E** containing 2,6-dibenzhydryl group delivered polyethylene with much higher molecular weight in the lowest yield when compared to **F** and **G** substituted by 2,4-dibenzhydryl group indicating that the requisite steric protection could largely inhibit chain termination as well as chain propagation leading to high molecular weight polymer inefficiently. In this work, the polyethylenes generated using **G**/MMAO are characteristic of polyethylene waxes displaying the lowest molecular weights (1.0–2.5 kg mol^−1^) and relatively narrower molecular weight distributions (1.92–2.27) among these four chromium catalysts in Figure 8. Therefore, **G**/MMAO shows great promise for potential industrial applications in the production of low molecular weight highly linear polyethylene waxes [51,75].

## 3. Materials and Methods

### 3.1. General Considerations

The air- and moisture-sensitive compounds were synthesized and handled under nitrogen atmosphere using standard Schlenk techniques. Prior to use, toluene was refluxed over sodium under nitrogen for 10h. The cocatalysts, methylaluminoxane (MAO, 1.46 M Al solution in toluene) and modified methylaluminoxane (MMAO, 2.00 M Al solution in n-heptane), were purchased from Albemarle Corp. (Baton Rouge, LA, USA). High-purity ethylene was purchased from Beijing Yanshan Petrochemical Co. (Beijing, China) and used as received. Other reagents were purchased from Aldrich (Beijing, China), Acros (Beijing, China) or Beijing Chemicals (Beijing, China). A Bruker Avance III 400 HD instrument (Bruker, Fällanden, Switzerland) was used to record the ^1^H- and ^13^C-NMR spectra of compounds and ligands at ambient temperature using TMS as an internal standard. The ^1^H- and ^13^C-NMR spectra of the resultant polyethylene were recorded on a Bruker DMX 300 MHz instrument (Bruker, Fällanden, Switzerland) at 100 °C using the deuterated 1,1,2,2-tetrachloroethane as the deuterium reagent. IR spectra were conducted on a System 2000 FT-IR spectrometer (Perkin-Elmer, Waltham, MA, USA) and elemental analysis (C, H, and N) was carried out using a Thermo Flash Smart EA microanalyzer (Thermo Fisher Scientific, Waltham, MA, USA). The molecular weight (M_w_) and molecular weight distributions (M_w_/M_n_) of resultant polyethylenes were determined by the Agilent PL-GPC220 GPC/SEC high-temperature system at 150 °C with 1,2,4-trichlorobenzene (TCB) as eluent with a flow rate of 1.0 mL min^−1^. Additionally, the melting point of polyethylene was measured from the fourth scanning run by the PerkinElmer TA-Q2000 differential scanning calorimetry (DSC) analyzer (TA Instruments, New Castle, DE, USA) under a nitrogen atmosphere. 

### 3.2. Synthesis of 2-Acetyl-6-(1-(2,4-dibenzhydryl-6-fluorophenylimino)ethyl)pyridine *(**1**)* and Ligands ***L1**–**L5***

#### 3.2.1. Synthesis of 2-Acetyl-6-(1-(2,4-dibenzhydryl-6-fluorophenylimino)ethyl)pyridine (**1**)

The catalytic amount *p*-toluenesulfonic acid (20% by mol) was added to the mixed suspension of 2,6-diacetylpyridine (4.08 g, 25 mmol) and 2,4-dibenzhydryl-6-fluoroaniline (11.09 g, 25 mmol) in *ortho*-xylene (150 mL). After 10 h stirring at refluxing temperature, the reaction mixture was filtered in the hot condition and all the volatile solvent was removed by a vacuum pump. Subsequently, the impurities were removed by basic alumina column chromatography using petroleum ether/ethyl acetate (25/1 *v/v*) as an eluent, yielding product **1** as a light-yellow powder (4.12 g, 28%). Anal. Calcd. for C_41_H_33_FN_2_O (588.73): H, 5.65; C, 83.65; N, 4.76. Found: H, 5.76; C, 83.47; N, 4.77. ^1^H-NMR (CDCl_3_, 400 MHz, TMS): δ 8.12 (d, *J* = 8.0 Hz, 2H, Py-H_m_), 7.88 (t, *J* = 7.6 Hz, 1H, Py-H_p_), 7.81 (s, 2H, aryl-H), 7.28-7.21 (m, 12H, aryl-H), 7.12-6.96 (m, 8H, aryl-H), 5.27 (s, 2H, CHPh_2_), 2.63 (s, 3H, O=CCH_3_), 1.08 (s, 3H, N=CCH_3_). ^13^C-NMR (CDCl_3_, 100 MHz, TMS): δ 199.8, 169.3, 153.9, 153.6, 152.6, 143.5, 141.7, 140.5, 136.3, 133.6, 129.6, 129.1, 128.2, 126.6, 126.7, 124.5, 123.6, 122.9, 52.1, 25.2, 17.2. FT-IR (cm^−1^): 3026 (w), 2927 (m), 1698 (*ν*_C=O_, s), 1655 (*ν*_C=N_, s), 1579 (m), 1513 (s), 1491 (m), 1447 (s), 1357 (s), 1324 (s), 1229 (s), 1152 (w), 1117 (w), 1079 (s), 1027 (m), 996 (w), 912 (m), 812 (s), 766 (w), 695 (s).

#### 3.2.2. Synthesis of 2-(1-(2,4-Dibenzhydryl-6-fluorophenylimino)ethyl)-6-(1-(2,6-dimethylphenyl-imino)ethyl)pyridine (**L1**)

In a two neck round bottom flask, the solution of 2-acetyl-6-(1-(2,4- dibenzhydryl-6-fluorophenylimino)ethyl)pyridine (2.06 g, 3.50 mmol) and *p*-toluenesulfonic acid (20% by mol) in *ortho*-xylene (40 mL) was added and refluxed at 145 °C. Subsequently, 2,6-dimethylaniline (0.42 g, 3.50 mmol) was added dropwise into the reaction solution. After 10 h, all the volatile solvent was removed by a vacuum pump and the residue was purified by basic alumina column chromatography using petroleum ether/ethyl acetate (125/1) as an eluent affording **L1** as a light-yellow powder (0.44 g, 18%). Anal. Calcd. for C_49_H_42_FN_3_ (691.89): H, 6.12; C, 85.06; N, 6.07. Found: H, 6.26; C, 84.80; N, 5.87. ^1^H-NMR (CDCl_3_, 400 MHz, TMS): δ 8.46 (d, *J* = 8.0 Hz, 1H, Py-H_m_), 8.35 (d, *J* = 7.6 Hz, 1H, Py-H_m_), 7.90 (t, *J* = 16.0 Hz, 1H, Py-H_p_), 7.30–6.79 (m, 23H, Ar-H), 6.77 (d, *J* = 11.2 Hz, 1H, Ar-H), 6.63 (s, 1H, Ar-H), 5.59 (s, 1H, CHPh_2_), 5.42 (s, 1H, CHPh_2_), 2.19 (s, 3H, N=CCH_3_), 2.08 (s, 6H, 2 × CH_3_), 1.87 (s, 3H, N=CCH_3_). ^13^C-NMR (CDCl_3_, 100 MHz, TMS): δ 170.93, 167.22, 155.03, 154.88, 151.94, 149.52, 148.74, 143.69, 142.56, 140.03, 139.97, 137.44, 136.72, 135.01, 134.87, 129.45, 129.29, 128.31, 128.17, 127.89, 126.37, 126.19, 125.44, 123.02, 122.43, 122.23, 114.86, 114.64, 56.16, 52.10, 17.96, 16.81, 16.79, 16.42. FT-IR (cm^−1^): 3027 (w), 2937 (w), 1643 (ν_C=N_, s), 1568 (w), 1496 (w), 1454 (s), 1365 (s), 1328 (w), 1298 (w), 1250 (s), 1207 (m), 1121 (s), 1030 (w), 857 (w), 765 (s).

#### 3.2.3. Synthesis of 2-(1-(2,4-Dibenzhydryl-6-fluorophenylimino)ethyl)-6-(1-(2,6-diethylphenylimino) ethyl)pyridine (**L2**)

Based on the procedure and half molar ratio that were used for the synthesis of **L1**, **L2** was prepared as a light-yellow powder (0.23 g, 18%). Anal. Calcd. for C_51_H_46_FN_3_ (719.95): H, 6.44; C, 85.08; N, 5.84. Found: H, 6.58; C, 84.75; N, 5.82. ^1^H-NMR (CDCl_3_, 400 MHz, TMS): δ 8.42 (d, *J* = 8.0 Hz, 1H, Py-H_m_), 8.32 (d, *J* = 7.6 Hz, 1H, Py-H_m_), 7.88 (t, *J* = 15.6 Hz, 1H, Py-H_p_), 7.28–6.97 (m, 23H, Ar-H), 6.75 (s, 1H, Ar-H), 6.60 (s, 1H, Ar-H), 5.59 (s, 1H, CHPh_2_), 5.43 (s, 1H, CHPh_2_), 2.51–2.23 (m, 4H, 2 × C*H*_2_CH_3_), 2.17 (s, 3H, N=CCH_3_), 1.85 (s, 3H, N=CCH_3_), 1.20–1.11 (m, 6H, 2 × CH_2_C*H*_3_). ^13^C-NMR (CDCl_3_, 100 MHz, TMS): δ 169.92, 165.90, 154.00, 153.84, 146.74, 142.64, 141.52, 138.98, 138.91, 136.38, 135.70, 133.97, 130.15, 128.41, 128.25, 127.26, 127.13, 125.33, 125.15, 124.89, 122.27, 121.35, 121.16, 113.81, 113.60, 55.11, 51.01, 23.55, 15.74, 12.69. FT-IR (cm^−1^): 3024 (w), 2930 (w), 1638 (ν_C=N_, s), 1567 (w), 1492 (w), 1449 (s), 1365 (s), 1325 (w), 1296 (w), 1243 (s), 1199 (m), 1118 (s), 1028 (w), 854 (w), 764 (s).

#### 3.2.4. Synthesis of 2-(1-(2,4-Dibenzhydryl-6-fluorophenylimino)ethyl)-6-(1-(2,6-diisopropylphenyl imino)ethyl)pyridine (**L3**)

Based on the procedure and half molar ratio that were used for the synthesis of **L1**, **L3** was prepared as a light-yellow powder (0.20 g, 15%). Anal. Calcd. for C_53_H_50_FN_3_ (748.00): H, 6.74; C, 85.10; N, 5.62. Found: H, 6.92; C, 84.96; N, 5.55. ^1^H-NMR (CDCl_3_, 400 MHz, TMS): δ 8.42 (d, *J* = 8.0 Hz, 1H, Py-H_m_), 8.32 (d, *J* = 8.0 Hz, 1H, Py-H_m_), 7.88 (t, *J* = 15.6 Hz, 1H, Py-H_p_), 7.28–6.76 (m, 23H, Ar-H), 6.75 (s, 1H, Ar-H), 6.61 (s, 1H, Ar-H), 5.59 (s, 1H, Ar-H), 5.43 (s, 1H, Ar-H), 2.79–2.72 (m, 2H, C*H*(CH_3_)_2_), 2.19 (s, 3H, N=CCH_3_), 1.85 (d, *J* = 12.0 Hz, 3H, N=CCH_3_), 1.20–1.14 (m, 12H, CH(C*H*_3_)_2_). ^13^C-NMR (CDCl_3_, 100 MHz, TMS): δ 169.93, 165.92, 154.01, 153.88, 145.44, 142.65, 141.44, 136.38, 135.69, 134.77, 133.85, 128.41, 128.25, 127.27, 127.13, 125.33, 125.15, 122.53, 121.96, 121.33, 121.20, 113.81, 113.60, 55.12, 51.04, 27.26, 22.18, 21.87, 16.01, 15.79. FT-IR (cm^−1^): 3024 (w), 2922 (w), 1643 (ν_C=N_, s), 1571 (w), 1493 (w), 1453 (s), 1361 (s), 1321 (w), 1300 (w), 1254 (s), 1205 (m), 1122 (s), 1032 (w), 849 (w), 765 (s).

#### 3.2.5. Synthesis of 2-(1-(2,4-Dibenzhydryl-6-fluorophenylimino)ethyl)-6-(1-(mesitylimino)ethyl) pyridine (**L4**)

Based on the procedure and half molar ratio that were used for the synthesis of **L1**, **L4** was prepared as a light-yellow powder (0.23 g, 20%). Anal. Calcd. for C_50_H_44_FN_3_ (705.92): H, 6.28; C, 85.07; N, 5.95. Found: H, 6.52; C, 84.74; N, 5.79. ^1^H-NMR (CDCl_3_, 400 MHz, TMS): δ 8.42 (d, *J* = 8.0 Hz, 1H, Py-H_m_), 8.31 (d, *J* = 7.6 Hz, 1H, Py-H_m_), 7.87 (t, *J* = 15.6 Hz, 1H, Py-H_p_), 7.28–6.89 (m, 22H, Ar-H), 6.76 (d, *J* = 10.8 Hz, 1H, Ar-H), 6.60 (s, 1H, Ar-H), 5.59 (s, 1H, Ar-H), 5.43 (s, 1H, CHPh_2_), 2.30 (s, 3H, Ar-CH_3_), 2.16 (s, 3H, N=CCH_3_), 2.01 (s, 6H, 2 × CH_3_), 1.84 (s, 3H, N=CCH_3_). ^13^C-NMR (CDCl_3_, 100 MHz, TMS): δ 169.91, 166.36, 154.12, 153.80, 150.90, 148.48, 145.19, 142.65, 141.52, 138.97, 138.91, 136.41, 135.64, 133.98, 133.84, 131.16, 128.40, 128.25, 127.52, 127.26, 127.13, 125.33, 125.14, 124.23, 121.31, 121.18, 113.81, 113.59, 55.11, 51.03, 19.71, 16.84, 15.76, 15.74, 15.32. FT-IR (cm^−1^): 3022 (w), 2911 (w), 1640 (ν_C=N_, s), 1567 (w), 1493 (w), 1472 (s), 1365 (s), 1327 (w), 1297 (w), 1255 (s), 1217 (m), 1119 (s), 1028 (w), 854 (w), 738 (s).

#### 3.2.6. Synthesis of 2-(1-(2,4-Dibenzhydryl-6-fluorophenylimino)ethyl)-6-(1-(2,6-diethyl-4-methyl- phenyl imino)ethyl)pyridine (**L5**)

Based on the procedure and half molar ratio that were used for the synthesis of **L1, L5** was prepared as a light-yellow powder (0.19 g, 15%). Anal. Calcd. for C_52_H_48_FN_3_ (733.98): H, 6.59; C, 85.09; N, 5.73. Found: H, 6.53; C, 84.80; N, 5.80. ^1^H-NMR (CDCl_3_, 400 MHz, TMS): δ 8.42 (d, *J* = 8.0 Hz, 1H, Py-H_m_), 8.33 (d, *J* = 7.6 Hz, 1H, Py-H_m_), 7.88 (t, *J* = 15.6 Hz, 1H, Py-H_p_), 7.29–6.94 (m, 22H, Ar-H), 6.76 (d, *J* = 10.4 Hz, 1H, Ar-H), 6.62 (s, 1H, Ar-H), 5.60 (s, 1H, CHPh_2_), 5.44 (s, 1H, CHPh_2_), 2.44-2.27 (m, 7H, 2 × C*H*_2_CH_3_, N=CCH_3_), 2.18 (s, 3H, N=CCH_3_), 1.85 (s, 3H, CH_3_), 1.14 (m, 6H, 2 × CH_2_C*H*_3_). ^13^C- NMR (CDCl_3_, 100 MHz, TMS): δ 169.95, 166.07, 154.13, 153.79, 150.89, 148.47, 144.22, 142.64, 141.52, 138.96, 138.89, 136.39, 135.65, 133.99, 133.85, 131.37, 130.04, 128.40, 128.24, 127.26, 127.12, 125.63, 125.32, 125.14, 121.26, 121.14, 113.80, 113.59, 55.10, 51.03, 23.54, 19.98, 15.77, 15.74, 15.67, 12.81. FT-IR (cm^−1^): 3026 (w), 2932 (w), 1638 (ν_C=N_, s), 1566 (w), 1493 (w), 1455 (s), 1365 (s), 1325 (w), 1296 (w), 1261 (s), 1207 (m), 1148 (s), 1026 (w), 855 (w), 739 (s).

### 3.3. Synthesis of Chromium Complexes ***Cr1**–**Cr5***

#### 3.3.1. Synthesis of 2-(1-(2,4-Dibenzhydryl-6-fluorophenylimino)ethyl)-6-(1-(2,6-dimethylphenyl imino)ethyl)pyridylchromium(III) chloride (**Cr1**)

CrCl_3_·3THF (0.07 g, 0.20 mmol) was added to the dichloromethane solution (10 mL) of 2-(1-(2,4-dibenzhydryl-6-fluorophenylimino)ethyl)-6-(1-(2,6-dimethylphenylimino)ethyl)pyridine (0.14 g, 0.20 mmol). This mixture was stirred at room temperature for 10 h giving a green suspension. Excess of diethyl ether (20 mL) was poured into the concentrated reaction mixture and the resulting precipitate was collected by filtration, washed with diethyl ether (3 × 5 mL) and dried under reduced pressure to give **Cr1** as green powder (0.14 g, 83%). Anal. calcd. for C_49_H_42_Cl_3_CrFN_3_ (850.24): H, 4.98; C, 69.22; N, 4.94; Found: H, 4.99; C, 68.97; N, 5.00. FT-IR (cm^−1^): 3062 (m), 3024 (m), 2959 (m), 2916 (m), 1694 (w), 1614 (m, v_C=N_), 1576 (m), 1492 (m), 1472 (m), 1448 (m), 1426 (w), 1369 (w), 1323 (w), 1268 (m), 1214 (m), 1174 (m), 1095 (m), 1035 (m), 999 (m), 913 (w), 847 (w), 814 (m), 774 (m), 743 (m), 699 (s), 658 (w).

#### 3.3.2. Synthesis of 2-(1-(2,4-Dibenzhydryl-6-fluorophenylimino)ethyl)-6-(1-(2,6-dimethylphenyl imino)ethyl)pyridylchromium(III) chloride (**Cr2**)

Using similar synthetic procedure and molar ratio described for the synthesis of **Cr1**, **Cr2** was prepared by reacting 2-(1-(2,4-dibenzhydryl-6-fluorophenylimino)ethyl)-6-(1-(2,6-diethylphenyl imino)ethyl)pyridine (0.14 g, 0.20 mmol) with CrCl_3_·3THF (0.07 g, 0.20 mmol) and collected as a light green powder (0.14 g, 80%). Anal. calcd. for C_51_H_46_Cl_3_CrFN_3_ (878.29): H, 5.28; C, 69.74; N, 4.78; Found: H, 5.28; C, 69.49; N, 4.88. FT-IR (cm^−1^): 3058 (m), 3020 (m), 2965 (m), 2917 (m), 1697 (w), 1614 (m, ν_C=N_), 1576 (m), 1494 (m), 1472 (m), 1448 (m), 1426 (m), 1369 (m), 1323 (w), 1303 (w), 1267 (w), 1214 (w), 1181 (w), 1095 (m), 1035 (m), 996 (m), 814 (m), 774 (m), 743 (m), 699 (s), 661(w).

#### 3.3.3. Synthesis of 2-(1-(2,4-Dibenzhydryl-6-fluorophenylimino)ethyl)-6-(1-(2,6-dimethylphenyl imino)ethyl)pyridylchromium(III) chloride (**Cr3**)

Using similar synthetic procedure and molar ratio described for the synthesis of **Cr1**, **Cr3** was prepared by reacting 2-(1-(2,4-dibenzhydryl-6-fluorophenylimino)ethyl)-6-(1-(2,6-diisopropyl phenylimino)ethyl)pyridine (0.15 g, 0.20 mmol) with CrCl_3_·3THF (0.07 g, 0.20 mmol) and collected as a light green powder (0.15 g, 83%). Anal. calcd. for C_53_H_50_Cl_3_CrFN_3_ (906.35): H, 5.56; C, 70.24; N, 4.64; Found: H, 5.67; C, 69.99; N, 4.76. FT-IR (cm^−1^): 3058 (m), 3031 (m), 2966 (m), 2914 (m), 1690 (w), 1618 (m, ν_C=N_), 1576 (m), 1514 (w), 1494 (m), 1471 (m), 1448 (m), 1426 (w), 1369 (w), 1326 (w), 1267 (m), 1217 (w), 1178 (m), 1098 (m), 1036 (m), 996 (m), 916 (w), 880 (w), 843 (w), 814 (w), 781 (m), 743 (m), 699 (s), 658 (w).

#### 3.3.4. Synthesis of 2-(1-(2,4-Dibenzhydryl-6-fluorophenylimino)ethyl)-6-(1-(2,6-dimethylphenyl imino)ethyl)pyridylchromium(III) chloride (**Cr4**)

Using similar synthetic procedure and molar ratio described for the synthesis of **Cr1**, **Cr4** was prepared by reacting 2-(1-(2,4-dibenzhydryl-6-fluorophenylimino)ethyl)-6-(1-(mesitylimino) ethyl)pyridine (0.14 g, 0.20 mmol) with CrCl_3_·3THF (0.07 g, 0.20 mmol) and collected as a green powder (0.15 g, 87%). Anal. calcd. for C_50_H_44_Cl_3_CrFN_3_ (864.27): H, 5.13; C, 69.49; N, 4.86; Found: H, 5.17; C, 69.41; N, 4.91. FT-IR (cm^−1^): 3058 (m), 3024 (m), 2969 (m), 2917 (m), 1697 (w), 1614 (m, ν_C=N_), 1576 (s), 1492 (m), 1469 (m), 1448 (m), 1426 (m), 1373 (m), 1330 (m), 1270 (m), 1217 (w), 1174 (w), 1098 (m), 1039 (m), 996 (m), 916 (w), 847 (w), 814 (m), 777 (w), 743 (m), 699 (s), 661 (w).

#### 3.3.5. Synthesis of 2-(1-(2,4-Dibenzhydryl-6-fluorophenylimino)ethyl)-6-(1-(2,6-dimethylphenyl imino)ethyl)pyridylchromium(III) chloride (**Cr5**)

Using similar synthetic procedure and molar ratio described for the synthesis of **Cr1**, **Cr5** was prepared by reacting 2-(1-(2,4-dibenzhydryl-6-fluorophenylimino)ethyl)-6-(1-(2,6-diethyl-4-methyl- phenylimino)ethyl)pyridine (0.15 g, 0.20 mmol) with CrCl_3_·3THF (0.07 g, 0.20 mmol) and collected as a light green powder (0.15 g, 84%). Anal. calcd. for C_52_H_48_Cl_3_CrFN_3_ (892.32): H, 5.42; C, 69.99; N, 4.71; Found: H, 5.59; C, 70.01; N, 4.79. FT-IR (cm^−1^): 3062 (m), 3024 (m), 2965 (m), 2917 (m), 1690 (w), 1611 (m, ν_C=N_), 1576 (s), 1494 (m), 1472 (m), 1448 (m), 1426 (m), 1370 (m), 1267 (m), 1214 (m), 1085 (m), 1036 (m), 996 (m), 848 (w), 810 (m), 774 (w), 743 (m), 699 (s), 655 (w).

### 3.4. X-ray Crystallographic Studies

Single-crystals of **Cr2** and **Cr4** suitable for X-ray diffraction analysis were obtained by slow diffusion of *n*-heptane into dichloromethane solution at room temperature. X-ray study was carried out on a MM007HF single crystal diffractometer with Confocal-monochromatized Mo-Kα radiation (Rigaku, Tokyo, Japan) (λ = 0.71073 Å) at 169.99(10) or 170.00(15) K, and cell parameters were obtained by the global refinement of the positions of all collected reflections. The structures were solved by direct methods and refined by full-matrix least-squares on *F*^2^. Intensities were corrected for Lorentz and polarization effects and empirical absorption. All the hydrogen atoms were placed in calculated positions. Structure solution and refinement were performed by using the SHELXT (Sheldrick, 2015, Göttingen, Germany) [76,77]. During the structural refinement, the disordered solvent was squeezed (**Cr2** and **Cr4**) with PLATON software (Utrecht University, Utrecht, Netherlands) [78,79]. Details of the X-ray structure determinations and refinements were provided in Table 6. Electronic Supporting Information (ESI) available: CCDC 2002415 and 2002416 contain the Supporting crystallographic data for complexes **Cr2** and **Cr4**. These data can be obtained free of charge from The Cambridge Crystallographic Data Centre via www.ccdc.cam.ac.uk/data_request/cif.

### 3.5. Ethylene Polymerization Procedures

#### 3.5.1. Ethylene Polymerization at 1 Atmosphere Ethylene Pressure

The precatalyst **Cr4** (1.9 mg, 2.0 μmol) was added to a Schlenk vessel which was equipped with a stirrer, followed by freshly distilled toluene (30 mL). When the chromium catalysts were completely dissolved in the toluene, the required amount of co-catalyst was then added by syringe. Under 1 atm of ethylene pressure, the reaction mixture kept stirring at the designated reaction temperature for 30 min. After reaction, the mixture was quenched with 10% hydrochloric acid in ethanol. The obtained polymer was washed with ethanol and dried under reduced pressure at 80 °C and weighed.

#### 3.5.2. Ethylene Polymerization at 5/10 Atmosphere Ethylene Pressure

The high-pressure polymerization reactions were carried out in a stainless-steel autoclave (250 mL) equipped with a mechanical stirrer, a temperature controller and an ethylene pressure control system. Freshly distilled toluene (25 mL) and toluene solution with chromium complex (50 mL) were successively injected into the autoclave when the designated reaction temperature reached. Then the required amount of co-catalyst was injected and more toluene (25 mL) was introduced to complete the addition. The autoclave was immediately pressurized to the designated ethylene pressure and the stirring commenced at the same time. When the reaction time was up, stop stirring and cool the reactor. The ethylene pressure was vented and 10% hydrochloric acid in ethanol was used to quenched the reaction mixture. The obtained polymer was washed with ethanol and dried under reduced pressure at 80 °C and weighed.

## 4. Conclusions

A series of *ortho*-fluorinated 2-[1-(2,4-dibenzhydryl-6-fluorophenylimino)ethyl]-6-[1-(aryl- imino)ethyl]pyridylchromium (III) chloride complexes has been synthesized in good yield and fully characterized including the molecular structure of complex **Cr2** and **Cr4** using-crystal X-ray diffraction. In the presence of MAO or MMAO, complexes **Cr1**–**Cr5** showed exceptionally good performance toward ethylene polymerization and produced highly linear polyethylene waxes. In general, the MMAO-activated chromium catalysts displayed higher activities than that seen earlier with **Cr**/MAO highlighting the effect of cocatalyst type on catalytic performance. Moreover, the activity of **Cr4**/MMAO was especially outstanding as a result of combination effects of steric and electronic properties and reached at 20.14 × 10^6^ g (PE) mol^−1^ (Cr) h^−1^ which was much higher than that of previously reported chromium analogs. This work further illustrates the systematic modification in the steric and electronic substituents of complexes providing a way to improve the catalyst performance and polymer microstructure.

## Figures and Tables

**Table 1:** Selected bond lengths and angles for **Cr2** and **Cr4**.

	Cr2	Cr4		Cr2	Cr4
bond lengths (Å)			bond angles (°)		
Cr1–Cl1	2.2900 (18)	2.2757 (12)	Cl1–Cr1–N1	174.81 (17)	176.21 (10)
Cr1–Cl2	2.2788 (19)	2.3295 (12)	Cl1–Cr1–N2	107.04 (15)	105.71 (9)
Cr1–Cl3	2.3217 (19)	2.3278 (12)	Cl1–Cr1–N3	99.53 (14)	99.22 (11)
Cr1–N1	2.003 (5)	1.985 (3)	Cl2–Cr1–N1	91.48 (15)	82.50 (10)
Cr1–N2	2.157 (5)	2.132 (3)	Cl2–Cr1–N2	89.18 (15)	88.62 (9)
Cr1–N3	2.133 (5)	2.136 (3)	Cl2–Cr1–N3	86.33 (14)	89.58 (10)
C2–N2	1.290 (8)	1.297 (5)	Cl3–Cr1–N1	85.08 (15)	87.23 (10)
C8–N3	1.299 (7)	1.281 (6)	Cl3–Cr1–N2	91.29 (15)	88.38 (9)
bond angles (°)			Cl3–Cr1–N3	91.61 (14)	88.98 (10)
Cl1–Cr1–Cl2	92.31 (8)	96.57 (5)	N1–Cr1–N2	76.5 (2)	77.97 (13)
Cl1–Cr1–Cl3	91.04 (8)	93.74 (4)	N1–Cr1–N3	77.2 (2)	77.12 (14)
Cl2–Cr1–Cl3	176.31 (8)	169.69 (5)	N2–Cr1–N3	153.20 (19)	155.05 (14)

**Table 2:** Ethylene polymerization studies with **Cr4**/MAO ^a^.

Entry	Cat.	Al:Cr	*T*, °C	*t*, min	PE, g	Activity ^b^	*M*_w_^c^	*M*_w_/*M*_n_^c^	*T*_m_^d^, °C
1	**Cr4**	2000	30	30	1.08	1.08	1.05	1.68	120.6
2	**Cr4**	2500	30	30	1.97	1.97	1.11	1.71	121.1
3	**Cr4**	3000	30	30	3.27	3.27	1.15	1.46	122.0
4	**Cr4**	3250	30	30	5.14	5.14	1.17	1.79	122.8
5	**Cr4**	3500	30	30	5.46	5.46	1.22	1.89	122.9
6	**Cr4**	3750	30	30	4.78	4.78	1.09	1.69	122.7
7	**Cr4**	4000	30	30	2.88	2.88	1.04	1.52	122.5
8	**Cr4**	3500	40	30	5.64	5.64	1.48	1.94	122.0
9	**Cr4**	3500	50	30	7.52	7.52	1.51	2.09	122.5
10	**Cr4**	3500	60	30	10.03	10.03	1.61	2.25	123.5
11	**Cr4**	3500	70	30	6.24	6.24	1.44	2.01	121.6
12	**Cr4**	3500	60	05	1.66	9.96	1.02	1.67	120.8
13	**Cr4**	3500	60	15	4.30	8.60	1.18	1.72	121.2
14	**Cr4**	3500	60	45	10.24	6.83	1.61	2.16	121.1
15	**Cr4**	3500	60	60	10.54	5.27	1.67	2.18	120.7
16 ^e^	**Cr4**	3500	60	30	4.88	4.88	0.98	1.86	121.3
17 ^f^	**Cr4**	3500	60	30	Trace	-	-	-	-

^a^ General conditions: 2 µmol of **Cr4**, 10 atm of ethylene, 100 mL of toluene; ^b^ 10^6^ g (PE)·mol^−1^(Cr)·h^−1^; ^c^ M_w_: in kg mol^−1^, determined by GPC; ^d^ Determined by Differential Scanning Calorimetry (DSC); ^e^ 5 atm of ethylene; ^f^ 1 atm of ethylene.

**Table 3:** Ethylene polymerization with **Cr1**–**Cr5**/MAO ^a^.

Entry	Precatalyst	PE, g	Activity ^b^	*M*_w_^c^	*M*_w_/*M*_n_^c^	*T*_m_^d^, °C
1	**Cr1**	8.28	8.28	1.01	1.62	121.8
2	**Cr2**	6.86	6.86	1.74	1.99	122.3
3	**Cr3**	6.16	6.16	1.78	2.03	122.9
4	**Cr4**	10.03	10.03	1.61	2.25	123.5
5	**Cr5**	7.27	7.27	2.23	2.22	125.2

^a^ General conditions: 2 µmol of **Cr**, 10 atm of ethylene, 100 mL of toluene, Al/Cr = 3500, T = 60 °C, 30 min; ^b^ 10^6^ g (PE)·mol^−1^(Cr)·h^−1^; ^c^ M_w_: in kg mol^−1^, determined by GPC; ^d^ Determined by DSC.

**Table 4:** Ethylene polymerization studies with **Cr4**/MMAO ^a^.

Entry	Cat.	Al:Cr	*T*, °C	*t*, min	PE, g	Activity ^b^	*M*_w_^c^	*M_w_*/*M*_n_^c^	*T*_m_^d^, °C
1	**Cr4**	2000	30	30	5.18	5.18	0.77	1.71	119.9
2	**Cr4**	2500	30	30	7.44	7.44	1.09	2.01	120.0
3	**Cr4**	3000	30	30	12.14	12.14	1.27	2.14	120.9
4	**Cr4**	3500	30	30	15.53	15.53	1.36	1.91	121.0
5	**Cr4**	3750	30	30	17.51	17.51	1.38	1.95	121.7
6	**Cr4**	4000	30	30	20.14	20.14	1.43	1.97	122.4
7	**Cr4**	4250	30	30	13.27	13.27	1.05	1.89	120.8
8	**Cr4**	4500	30	30	11.14	11.14	0.78	1.62	120.5
9	**Cr4**	4000	20	30	13.28	13.28	0.89	1.76	120.3
10	**Cr4**	4000	40	30	17.84	17.84	1.20	2.07	122.0
11	**Cr4**	4000	50	30	13.63	13.63	1.07	1.80	121.5
12	**Cr4**	4000	60	30	11.76	11.76	1.06	1.73	120.4
13	**Cr4**	4000	30	05	3.03	18.18	0.67	1.39	119.1
14	**Cr4**	4000	30	15	9.52	19.04	0.73	1.52	120.3
15	**Cr4**	4000	30	45	22.31	14.87	1.48	1.88	120.4
16	**Cr4**	4000	30	60	22.79	11.39	1.55	2.42	120.7
17 ^e^	**Cr4**	4000	30	30	8.56	8.56	0.76	1.71	119.4
18 ^f^	**Cr4**	4000	30	30	Trace	-	-	-	-

^a^ General conditions: 2 µmol of **Cr4**, 10 atm of ethylene, 100 mL of toluene; ^b^ 10^6^ g (PE)·mol^−1^(Cr)·h^−1^; ^c^ M_w_: in kg mol^−1^, determined by GPC; ^d^ Determined by DSC; ^e^ 5 atm of ethylene; ^f^ 1 atm of ethylene.

**Table 5:** Ethylene polymerization with **Cr1**–**Cr5**/MMAO ^a^.

Entry	Precatalyst	PE, g	Activity ^b^	*M*_w_^c^	*M*_w_/*M*_n_^c^	*T*_m_^d^, °C
1	**Cr1**	8.93	8.93	1.01	1.92	120.4
2	**Cr2**	10.78	10.78	1.59	2.12	121.3
3	**Cr3**	7.59	7.59	1.80	2.01	123.5
4	**Cr4**	20.14	20.14	1.43	1.97	122.4
5	**Cr5**	12.67	12.67	2.49	2.27	125.0

^a^ General conditions: 2 µmol of **Cr**, 10 atm of ethylene, 100 mL of toluene, Al/Cr = 4000, T = 30 °C, 30 min; ^b^ 10^6^ g (PE)·mol^−1^(Cr)·h^−1^; ^c^ M_W_ in kg mol^−1^, determined by GPC; ^d^ Determined by DSC.

**Table 6:** Crystal data and structure refinement for **Cr2** and **Cr4**.

Complex	Cr2	Cr4
CCDC No.	2,002,415	2,002,416
Empirical formula	C51H46Cl3CrFN3	C50H44Cl3CrFN3
Formula weight	878.25	864.23
Temperature (K)	169.99 (10)	170.00 (15)
Wavelength (Å)	0.71073	0.71073
Crystal system	Monoclinic	Monoclinic
Space group	P21/c	P21/c
a (Å)	15.9706 (6)	27.1328 (5)
b (Å)	39.1059 (18)	27.2064 (4)
c (Å)	15.2664 (6)	17.0515 (3)
α (°)	90	90
β (°)	91.412 (4)	104.612 (2)
γ (°)	90	90
Volume (Å^3^)	9531.7 (7)	12180.1 (4)
Z	4	4
Dcalcd (g cm^−^^3^)	1.224	0.943
µ (mm^−^^1^)	3.832	2.922
F (000)	3656.0	3592.0
Crystal size (mm)	0.53 × 0.29 × 0.18	0.20 × 0.15 × 0.09
2θ Range (°)	4.52 to 151.222	4.678 to 151.134
Limiting indices	−19 ≤ h ≤ 19, −48 ≤ k ≤ 47, −19 ≤ l ≤ 18	−33 ≤ h ≤ 25, −32 ≤ k ≤ 34, −21 ≤ l ≤ 21
No. of rflns collected	78028	103413
No. unique rflns [R(int)]	18,707 [R_int_ = 0.0992, R_sigma_ = 0.0691]	24,269 [R_int_ = 0.0847, R_sigma_ = 0.0633]
Completeness to θ (%)	100%	100%
Data/restraints/parameters	18,707/81/1082	24,269/72/1055
The goodness of fit on *F*^2^	1.349	1.044
Final R indices [I > 2σ(I)]	R1 = 0.1094, wR2 = 0.3181	R1 = 0.0776, wR2 = 0.2220
R Indices (all data)	R1 = 0.1436, wR2 = 0.3634	R1 = 0.1155, wR2 = 0.2581
